# Sepsis: mechanisms of bacterial injury to the patient

**DOI:** 10.1186/s13049-019-0596-4

**Published:** 2019-02-14

**Authors:** Hayk Minasyan

**Affiliations:** Yerevan, Armenia

**Keywords:** Sepsis, Treatment, Antibacterial therapy, Antibiotics, Bactericidals, Bacteriostatics

## Abstract

In bacteremia the majority of bacterial species are killed by oxidation on the surface of erythrocytes and digested by local phagocytes in the liver and the spleen. Sepsis-causing bacteria overcome this mechanism of human innate immunity by versatile respiration, production of antioxidant enzymes, hemolysins, exo- and endotoxins, exopolymers and other factors that suppress host defense and provide bacterial survival. Entering the bloodstream in different forms (planktonic, encapsulated, L-form, biofilm fragments), they cause different types of sepsis (fulminant, acute, subacute, chronic, etc.). Sepsis treatment includes antibacterial therapy, support of host vital functions and restore of homeostasis. A bacterium killing is only one of numerous aspects of antibacterial therapy. The latter should inhibit the production of bacterial antioxidant enzymes and hemolysins, neutralize bacterial toxins, modulate bacterial respiration, increase host tolerance to bacterial products, facilitate host bactericidal mechanism and disperse bacterial capsule and biofilm.

## Introduction

Recently WHO has recognized sepsis as a Global Health Priority [1]. The true burden of sepsis remains unknown. The current estimates of 30 million episodes and 6 million deaths per year come from a systematic review that extrapolated from published national or local population estimates to the global population [2]. This estimate is based on data on hospital-treated sepsis in high-income countries and does not include statistics from the low- and middle-income countries (LMICs) where 87% of the world’s population lives. This lack of data is compounded by the fact that sepsis is treated as a “garbage code” in the Global Burden of Disease statistics, where most deaths due to sepsis are classified as being caused by the underlying infection [3]. Sepsis is associated with a mortality rate of 25–30% and mortality due to septic shock is 50–85% [4, 5]. Empiric antimicrobial therapy is the cornerstone of the treatment [6]. Current guidelines recommend starting antibiotic therapy within one hour of identification of septic shock [7]. Every hour delay is associated with a 6% rise in mortality [8, 9]. There are no prospective data that early broad-spectrum antibiotic therapy reduces mortality in severe sepsis, but prompt initiation of antimicrobial therapy remains important for suspected infections [10]. If the pathogen is resistant to antibiotic, early or late initiation of antibiotic therapy cannot improve the outcome. Inappropriateness of empirical antibiotic therapy can contribute to high level of mortality [11]. The crisis emerges of antibiotic resistance for microbial pathogens [12–14]. As a result, the treatment of sepsis becomes increasingly difficult. Numerous mechanisms of bacterial resistance are revealed and described in detail [15–18]. At the same time, some other causes of decreased effectiveness of antibacterial therapy in sepsis are less reported.

## Human innate immunity in sepsis

The pathogenesis of the sepsis syndrome is dependent on activation of the innate immune response. Innate immunity plays a direct role in the development of sepsis and is also crucial for the activation and modulation of later antigen-specific adaptive immune responses. The clinical manifestations of sepsis and the systemic inflammatory response syndrome (SIRS) can be attributed to components of the innate immune response [19].

At present at least five lines of innate immunity defense against sepsis-causing bacteria are identified. Bacteria usually first enter host tissues. If the tissue is injured and a hemorrhage is available, infection confronts blood clot and complement as the first line of host defense; otherwise antigen-presenting cells such as monocytes/macrophages play major roles as sentinels for first line alerts or as mediators that shape the adaptive immune response [20]. Once activated by microbial products, macrophages acquire microbicidal competence that usually leads to effective immunity [21]. However, several bacterial pathogens, first of all, sepsis-causing bacteria, have evolved mechanisms of inhibiting macrophages and host immune response.

Bacteria enter the bloodstream when they penetrate tissue barrier. In the bloodstream erythrocytes are the main bactericidal cells [22]. Entering the bloodstream, bacteria move with blood flow and become electrically charged because of the phenomenon of triboelectric (friction) charging. Bacterial cell wall is a negatively charged structure. The electro negativity (zeta (ζ) electrokinetic potential) of bacteria depends on bacterial physiological state whereas the triboelectric charge of bacteria is determined by the friction of bacteria with blood plasma, vessel walls and other cells. The triboelectric charge of bacteria considerably exceeds bacterial zeta potential and interacts with any nearby electric charge that is strong enough. Erythrocytes are the cells with very strong electric charge. Erythrocyte membrane properties (density, elasticity, deformability, flexibility, frictional resistance, etc.) and biconcave shape provide strong triboelectric charging during rubbing to other cells and vessel walls. The blood flow induces additional charging of erythrocytes dependent on the speed, kind (laminar, turbulent) of blood flow and blood viscosity. Triboelectrically charged erythrocyte membrane immediately attracts and fixes nearby floating bacteria. Microbial proteases and pathogen-associated molecular patterns irritate erythrocyte membrane and provoke rapid release of reactive oxygen species from hemoglobin to the surface of erythrocytes. The majority of bacteria are sensitive to oxidation and released reactive oxygen species (peroxides, superoxide, hydroxyl radical, singlet oxygen, etc.) rapidly kill bacteria on the surface of erythrocytes. As a result, bacteria are killed in the bloodstream rapidly: erythrocytes first attract and keep bacteria by electric charge, then bacteria on the surface of erythrocytes cause immediate release of oxygen from oxyhemoglobin to the surface of erythrocytes and finally released oxygen oxidizes and kills bacteria [22, 23]. Killed bacteria lose both triboelectric charge and zeta potential and are washed from erythrocyte surface out into blood plasma. Passing the liver and the spleen, killed bacteria are caught and digested by Kuppfer cells of the liver and lymphoid tissue macrophages of the spleen [24, 25].

Oxidation of bacteria on the surface of erythrocytes kills the majority of bacterial species, but sepsis-causing bacteria have evolved numerous mechanisms against oxidation that provide their survival in the bloodstream. These mechanisms are highly effective and even additional saturation of arterial blood by hyperbaric oxygen does not provide killing of sepsis-causing bacteria in the bloodstream.

The next line of host defense is intravascular coagulation that may cause disseminated intravascular coagulation. In the case of host survival, phagocytosis of hematogeniously disseminated bacteria in the tissues is the last line of innate immunity defense [24, 25].

## The mechanisms of bacterial survival in the tissues and the effectiveness of antibacterials

Host tissues are a hostile environment for bacterial pathogens. For survival, growth and proliferation bacteria have evolved different mechanisms of adaptation, particularly, production of a thick capsule, biofilm formation and switching into the L-form.

### Capsule production

Capsular polysaccharide (CPS) plays important biological role in nutrient uptake [26], protection against environmental stresses [27], biofilm formation [28], survival against phagocytosis or antibiotics; it is also an important virulence factor [29, 30].

Capsule is located immediately exterior to the murein (peptidoglycan) layer of gram-positive bacteria and the outer membrane (lipopolysaccharide layer) of gram-negative bacteria. All sepsis-causing bacteria (*Staphylococcus aureus, Streptococcus pneumoniae, Haemophilus influenzae, Neisseria meningitidis, Klebsiella pneumoniae, Escherichia coli,* group B streptococci, etc.) have polysaccharide capsules on their surface. Capsule considerably decreases the ability of antimicrobial agents to gain entry into the cell where the drug targets are located [31]. Bacteria with capsule show high resistance to antibiotics [32].

When bacteria are exposed to sub-inhibitory levels of antibiotics, resistance to other structurally and functionally unrelated antibiotics is also observed [33]. Exposure to sub-inhibitory antibiotic concentrations causes increased production of capsular polysaccharide in bacteria [34, 35]. Bacterial capsule provides antibacterial resistance by blocking the uptake of antibacterial agents [36].

### Switching into the L-form

The majority of antibacterials, particularly, bactericidal antibiotics, kill bacteria by inhibiting the growth of bacterial wall. The wall is an important target for antibiotics and fragments of the wall are recognized by innate immune receptors [37]. Bacterial wall is an essential structure for viability: it protects the cell protoplast from mechanical damage and from osmotic rupture. At the same time, it enables bacterial interior to interact with the surrounding milieu and to exchange both substances and information. The wall is also crucial for cell division [38].

Inhibition of bacterial cell wall synthesis can stimulate bacteria to switch into a wall-deficient state called the L-form. The L-form transition is available in a wide range of bacteria. Most bacterial species can be converted into L-forms by antibiotics that inhibit cell wall synthesis [39]. L-forms are completely resistant to wall-targeting antibiotics, such as penicillins and cephalosporins [40]. L-forms of group B *Neisseria meningitidis* may be produced by penicillin, methicillin, ampicillin, cephalothin, cyclo-serine, ristocetin, bacitracin and vancomycin. These L-forms may be propagated serially on medium containing each antibiotic, and all L-forms have similar growth, morphologic and fermentative properties [41]. L-forms of *P. aeruginosa* are resistant to carbenicillin, piperacillin, cetsulodin, apalcillin, gentamicin, streptomycin, dibekacin, polymyxin B and colistin which have a high activity to their parent forms [42].

L-forms cause a wide range of persistent or recurrent infections of the urinary, cardiovascular, cerebrospinal systems, respiratory, gastrointestinal, integumentary and reproductive systems [43]. L-form also may penetrate to the bloodstream causing L-form bacteremia and sepsis.

### Biofilm formation

The formation of biofilm is an adaptation of microbes to hostile environments [44]. Microbial biofilms is the most “defensive” life strategy that adopted by bacteria [45]. Biofilms protect the microbial community from external damage. Bacteria with “a biofilm background” avoid phagocytosis by naïve macrophages and often cause chronic infection [46]. Biofilms are accounting for over 80% of microbial infection in human body [47].

Bacterial biofilms are highly resistant to antibiotic treatment and immune responses. In comparison with planktonic cultures, biofilm formation leads to a large increase (up to 1000-fold) in resistance to antimicrobial agents [48]. Aggressive and intensive antibiotic treatment is usually helpful to control the exacerbations of chronic biofilm infections induced by dispersed bacteria and reduce the biofilms, but cannot eradicate the biofilm infections [49].

The adequate concentration of antibiotic for eradication of mature biofilm is difficult to reach in vivo [50].

## Planktonic bacteria in the tissues

Bacterial cell exhibit two types of growth mode: planktonic cell and sessile aggregate which is known as the biofilm. Antoni van Leeuwenhoek in 1673 described planktonic microorganisms. Much of the knowledge of microbiology is based on studying free-floating bacteria.

Sepsis-causing planktonic bacteria usually rapidly proliferate in the tissues. They exhibit different stages of population development that may include: a. lag phase; b. logarithmic (exponential) phase; c. stationary phase (host defense starts to inhibit bacterial growth); d. death phase (the host defense against the pathogen if effective), capsule production and transition to biofilm growth (the host defense against the pathogen is relatively effective) or the phase of active proliferation (the host defense is overcome by the pathogen).

Planktonic bacteria in the tissues are free-living organisms: they float in the tissue liquids. They are relatively hydrophilic, their capsule (polysaccharide glycocalyx) is thin and does not interfere bacterial cell metabolic exchange with the tissue liquids. Sepsis-causing planktonic bacteria may be either single-celled (*Escherichia coli, Acinetobacter baumanii, Salmonella enterica, Shigella dysenteriae, Pseudomonas aeruginosa, Proteus mirabilis, Serratia marcescens* and others) or two- and multiple-celled (*Neisseria meningitides, Klebsiella pneumonia, Streptococcus pneumonia, Staphylococcus aureus and others*) organisms. The latter are not single-celled even in laboratory cultures. The majority of single-celled planktonic bacteria are motile whereas two- and multiple-celled pathogens are not motile and float passively with the liquid flow. In the bloodstream motile bacteria are more triboelectrically charged than non motile microbes. Planktonic bacteria with active metabolism, growth and proliferation are most sensitive to antimicrobials [51].

Planktonic bacteria cause acute inflammation in the tissues. They stimulate vascular leakage and exudate production that provide aquatic media for their rapid proliferation and dissemination [52]. Early adequate bactericidal therapy may eradicate infection. The use of bacteriostatic drugs does not clear bacterial population and may have the same results as the use of bactericidal antimicrobials in bacteriostatic doses. Combined use of bactericidal and bacteriostatic antimicrobials is often counter indicated because in the presence of a bactericidal drug that alone is capable of clearing a bacterial population, the addition of a bacteriostatic drug may result in a decrease in killing rates and an increase in the number of survived bacteria. For example, combinations of 30S protein synthesis and cell wall biosynthesis inhibitors, 50S protein synthesis and gyrase inhibitors, and cell wall biosynthesis and folic acid synthesis inhibitors show antagonism [53]. On the other hand, the relevance of classifying antibiotics as bacteriostatic or bactericidal has been questioned due to the reliance of these categories on drug concentrations and the treated organisms [54]. The bacteriostatic/bactericidal classification system varies across organisms and even across drug concentrations and the interactions between drugs may similarly shift [53]. Antibiotic combination therapy remains an important option as a treatment strategy aimed at controlling the rise of resistance. At the same time, the combined use of antibacterial drugs may increase toxicity and side effects, besides, it is not always clear which drug in combination is really effective. Local microbial pattern based on site infection and pattern of antibiotics sensitivity test can be used as supporting data to optimize appropriateness of empirical antibiotics therapy in sepsis patients [55].

## Bacteria in the bloodstream

Bacteria may circulate in the bloodstream as planktonic (free-floating and inside erythrocytes) bacteria, encapsulated bacteria, biofilm fragments and L-form (free-floating and inside leukocytes). Bacteria can enter the bloodstream as a complication of infections (pneumonia, meningitis, peritonitis, urinary tract infections, etc.), during surgery, due to catheters, vascular prostheses, prosthetic cardiac valves, other medical devices and tissue engineering constructs and foreign bodies placed in the tissues, during intravenous drug abuse and others [56]. Transient bacteremia can result after dental procedures or brushing of teeth [57].

### Planktonic bacteria in the bloodstream

Planktonic bacteria usually enter the bloodstream from the tissues. After syringe invention, intravenous and intraarterial injections provide direct access to the bloodstream. In intravenous injection of contaminated material, infection bypasses extravascular compartment (the tissues) and directly enters the bloodstream. WHO estimates that 40% of the more than 16 billion injections administered worldwide annually involve reused, unsterilized syringes and needles, with rates of unsafe injections climbing to 70% in some countries. The reuse of injection equipment is responsible worldwide for 33% of new HBV infections, 42% of HCV infections, and 2% of new HIV infections [58]. Bacterial complications in contaminated intravenous infections are rare [59–61], whereas the development of viral infection is often and this is a paradox because dirty and re-used syringes always contain different bacterial species whereas viral contamination is rarer. This paradox may be explained by bactericidal effectiveness of bacteria killing by oxidation on the surface of erythrocytes [20–25]. This mechanism is effective regarding the majority of bacterial species excluding sepsis-causing bacteria. The latter survive oxidation on the surface of erythrocytes by production of anti oxidative enzymes (catalase, superoxide dismutase, glutathione peroxidase, etc.) and respiration adapted to high concentrations of active oxygen (Table 1).

**Table 1:** Microbiological features of sepsis causing bacteria that cause problems to host defense

	*Staphylococcus aureus*	*Coagulase-negative staph*	*Streptococcus pneumonia*	*Haemophilus influenza b*	*Neisseria meningitidis*	*Klebsiella pneumonia*	*Enterococcus faecalis*	*Acinetobacter baumanii*	*Escherichia coli*	*Salmonella enterica*	*Shigella dysenteriae*	*Citrobacter freundii*	*Serratia marcescens*	*Proteus mirabilis*	*Pseudomonas aeruginosa*	*Bacteroides fragilis*
Glutathione peroxidase	+	+	+	+	+	+	+	+	+	+	+	+	+	+	+	+
Catalase	+	+	+	+	+	+	+	+	+	+	+	+	+	+	+	+
Superoxide dismutase	+	+	+	+	+	+	+	+	+	+	+	+	+	+	+	+
Hemolysins	+	+	+	+	+	+	+	+	+	+	+	+	+	+	+	+
S-layer	+	+	+	+	+	+	+	+	+	+	+	+	+	+	+	+
Capsule	+	+	+	+	+	+	+	+	+	+	+	+	+	+	+	+
Slime layer	+	+	+	+	+	+	+	+	+	+	+	+	+	+	+	+
Biofilm	+	+	+	+	+	+	+	+	+	+	+	+	+	+	+	+
Respiration	FAN	FAN	FAN	FAN	AMA	FAN	FAN	A	FAN	FAN	FAN	FAN	FAN	FAN	FAN	OAN

Abbreviations: *FAN* Facultative anaerobic bacteria, *MA* Micro aerobic bacteria, *OAN* Obligate anaerobic bacteria, *A* Aerobic bacteria

Moreover, on the surface of erythrocytes all sepsis-causing bacteria produce hemolysins (α-hemolysin, β-hemolysin, γ-hemolysin) that form pores in phospholipid bilayer of erythrocytes [62, 63]. As a result, the bacteria may enter erythrocytes, survive and proliferate there using hemoglobin as a source of nutrition. Proliferating in erythrocytes, sepsis-causing bacteria form a bacterial reservoir that constantly disseminates the bloodstream and distant tissues.

### Encapsulated bacteria in the bloodstream

Bacterial capsule provides physical, chemical and immunologic shielding of bacteria [64]. In the bloodstream bacterial capsule increases bacterial virulence [65]. Capsule prevents triboelectric charging of bacteria and electrical attraction and fixation on the surface of erythrocytes [24]. As a consequence, encapsulated bacteria evade oxidation and killing on the surface of erythrocytes. Encapsulated bacteria also do not enter erythrocytes and cannot cause disseminated intravascular coagulation by provoking abundant release of oxygen from erythrocytes [23, 24].

Entering the bloodstream from the primary site of infection, encapsulated bacteria spread to distant organs and tissues by blood flow, causing metastatic local acute or chronic infection with or without biofilm formation. Acute or subacute septic bacterial endocarditis (endocarditis lenta) and acute or subacute septic arthritis may be examples of encapsulated bacteria dissemination to distant locations [66].

### Biofilm fragments in the bloodstream

Biofilm is the natural mode of bacterial growth in nature [46]. Being a survival strategy of bacteria, biofilms may grow in the primary focus of infection and provide dissemination of bacteria by the bloodstream. Biofilm may hematogenously spread both planktonic bacteria and biofilm fragments [67]. Another source of bacterial biofilms in the bloodstream is indwelling blood-contacting medical devices (arterial and venous catheters and prostheses, mechanical heart valves, etc.). Biofilms on indwelling medical devices may be composed of gram-positive or gram-negative bacteria or yeasts. Bacteria commonly isolated from these devices include the gram-positive *Enterococcus faecalis, Staphylococcus aureus, Staphylococcus epidermidis, Streptococcus viridans; and the gram-negative Escherichia coli, Klebsiella pneumoniae, Proteus mirabilis, and Pseudomonas aeruginosa*. Biofilms may initially be composed of single species, but longer exposures inevitably lead to multispecies biofilms [68–70].

### L-form bacteria in the bloodstream

L-form bacteria enter the bloodstream from the tissues after surviving treatment by wall-targeting antibacterials. L-form bacteria can persist in the tissues for an extended period of time mainly attached to macrophages. Being engulfed, L-forms are not digested and continue to persist within macrophages [71]. Since bacterial L-forms are lacking peptidoglycan, they do not trigger an innate immune response [72]. L-forms enter the bloodstream being inside leukocytes and also in form of free spheroplasts and protoplasts. L-forms are able to replicate by unusual modes and persist in the tissues for a long time [73]. Their ability to resist phagocytosis, escape immune surveillance and to integrate with host cell organelles in antigen complexes, could provoke immune-pathologic consequences and as a result L-forms are key players in persistence and expression of pathology in the human body [74]. In the bloodstream, free-floating spheroplasts and protoplasts are fragile and are destroyed during motion; they survive inside leukocytes, erythrocytes and platelets [75, 76]. Antibacterial medications poorly penetrate into blood cells [77, 78]. L-forms are released after decomposition of infected erythrocytes, leukocytes and platelets and engulfed by spleen and liver macrophages. L-forms may contaminate and proliferate in distant tissues that may be an auspicious media for L-forms [79]. Although L-forms bacteremia does not cause a condition with typical clinical signs of sepsis, they are potentially important pathogens associated with atypical, chronic or latent infection.

## The effectiveness of antibacterials in the bloodstream

In sepsis the effectiveness of antibacterials in the bloodstream is limited by different factors. The optimal strategies and the effectiveness of antibacterial treatment in sepsis depend on bacterial forms of sepsis-causing bacteria, types of sepsis, pathogen sensitivity to antibacterials, microbe respiration and metabolism, bacterial mechanisms to avoid or defeat host defense and others (Table 2).

**Table 2:** bacterial forms of sepsis-causing bacteria, types of sepsis and the optimal strategies of antibacterial treatment

Bacterial form	Types of sepsis	Sensitivity to antibacterials	Respiration, Metabolism	Defense against host	Treatment strategies
Planktonic	Sepsis Severe sepsis Septic shock Fulminant sepsis	High	High	Toxins Catalase SOD, GPX, Hemolysins…	Appropriate antibacterials Toxin production inhibition Toxin binding and removal Antioxidant enzyme inhibition Inhibition of hemolysins Removal by mechanical device
Encapsulated	Subacute sepsis Chronic sepsis Relapsing sepsis Indolent sepsis Latent sepsis Dormant sepsis	Moderate	Moderate	Capsular polysaccharides	Appropriate antibacterials Capsule production inhibition
Biofilm		Low	Low	Biofilm polymers	Appropriate antibacterials after using antibiofilm drugs
L-form		Very low	Very low	Entering host cells	Not available

Abbreviations: *SOD* Superoxide dismutase, *GPX* Glutathione peroxidase

Planktonic bacteria cannot grow and multiply in the bloodstream because they become triboelectrically charged during move in the blood flow and friction with blood cells and vessel walls [23]. Triboelectric charge inhibits metabolism of planktonic bacteria by blocking bacterial trans membrane exchange. As a result, bacteria cannot grow and proliferate and the effectiveness of antibacterial agents in the bloodsteam dramatically decreases. Moreover, sepsis-causing planktonic bacteria enter erythrocytes by producing hemolysins (that locally destroy erythrocyte membrane) and proliferate inside erythrocytes being protected against oxidation by synthesis of antioxidant enzymes. Planktonic bacteria inside erythrocytes are resistant against antibacterial agents because the latter poorly penetrate and accumulate inside erythrocytes.

Encapsulated bacteria and bacteria in biofilms are resistant to antibacterials, particularly, antibiotics because of low metabolism and polysaccharides that “isolate” bacteria from antibacterial agents. Being for bacteria chemical and electrical insulators, bacterial capsule decreases sensitivity to antimicrobials at least by two ways: (a) slowing down bacterial metabolism, growth and multiplication; (b) blocking the access of antimicrobials to bacterial cell. Encapsulated bacteria are resistant to high concentrations of antimicrobial drugs.

L-form bacteria (free and inside leukocytes) are resistant to wall-targeting antibiotics because of the absence of bacterial wall. Because of low metabolic rate, L-form bacteria may be insensitive or only slightly sensitive to antibacterials with other (than wall-targeting) mechanisms of action. Inside blood cells (erythrocytes, leukocytes, platelets), L-forms are resistant to high concentrations of antibacterials. Most antibacterials are not enough lipid-soluble for penetrating inside the blood cells; besides, serum proteins bind antibacterials [77, 78].

Bacteria within biofilms are highly resistant to antimicrobial agents because of slow growing [69], besides, biofilm exopolymers block the access of antimicrobials to bacteria [70]. At the same time, planktonic cells that are shed from virtually all mature biofilms, are generally susceptible to antibiotics. Planktonic bacteria released from the biofilm micro-colonies may cause bacteremia and sepsis. Many of the cells that detach from biofilms growing on native heart valves (resulting in endocarditis) or vascular catheters are in the form of matrix-enclosed biofilm fragments that are very resistant to antibiotics, and they usually circulate until they “jam” in a capillary bed [67]. Thus, the microorganisms in biofilms are difficult or impossible to treat with antimicrobial agents; detachment from the device may result in acute infection and sepsis.

## Different types of sepsis

When an infection surpasses local tissue containment, bacteria enter the bloodstream and cause bacteremia. Local infection may be the source of systematic leakage of bacteria, bacterial components and products of damaged tissue to the bloodstream. Bacteria can enter the bloodstream as planktonic bacteria, encapsulated bacteria, biofilm fragments, L-form bacteria.

In the bloodstream the majority of planktonic free floating bacteria are killed by oxidation on the surface of erythrocytes. Sepsis does not develop as long as this mechanism is effective. Sepsis-causing bacteria usually survive oxidation on the surface of erythrocytes, enter erythrocytes and proliferate there. They produce toxins that intoxicate host, besides, they provoke abundant release of oxygen from erythrocytes that causes disseminated intravascular coagulation, general hypoxia and multiple organ failure [24].

Encapsulated bacteria, biofilm fragments and L-forms have low metabolism and cause less aggressive infection. Exopolymer shielding (encapsulated bacteria, biofilm fragments) and the absence of bacterial wall (bacterial L-form) prevent triboelectric charging and they are not attracted and killed on the surface of erythrocytes. Encapsulated forms and biofilm fragments of exotoxin-producing bacteria produce relatively little amount of exotoxins because of low metabolic activity and insulation by exopolymers whereas endotoxin-producing bacteria may intoxicate the host after being decomposed by local macrophages in the liver and the spleen. L-form bacteria are inactive in the bloodstream. Non-planktonic forms of sepsis-causing bacteria may cause different types of sepsis (sepsis, subacute sepsis, chronic sepsis, latent sepsis, indolent sepsis, dormant sepsis etc.) [80, 81] and initiate a broad spectrum of pathologies starting from pyelonephritis [82], reactive arthritis [83], type II diabetes [84], carotid arterial plaques [85], coronary thrombosis [86], atherosclerosis [87, 88] and ending with recurrent bloodstream infections [89–91] and relapsing sepsis [92]. A primary focus of infection and/or metastatic foci may become a long-term source of bacteremia resistant to antibacterial medications.

## The problems of antibacterial therapy in sepsis

### Bacterial toxins

The mechanisms by which bacteria cause sepsis and septic shock involve bacterial factors (cell wall, secreted products) and host factors (susceptibility, primary (immune) response, secondary (tissue) response, etc.) [93]. Bacterial toxins allow the pathogen to modulate host defenses. The type of toxin plays a major role in the outcome of disease [94]. Exotoxins usually are produced by living bacteria whereas endotoxins are released by dying or dead microorganisms and as a result, prompt killing of bacteria contains some risks of rapid intoxication of the host [95]. In sepsis bacterial endotoxin triggers such serious complications as shock, adult respiratory distress syndrome, and disseminated intravascular coagulation. These events often occur when appropriate antimicrobial therapy has been instituted [96]. In some infections with bacteremia, antibiotic therapy can cause release of bacterial endotoxin-like products and cause a Jarisch–Herxheimer reaction [97]. It occurs after initiation of antibacterials in louse-borne relapsing fever, tick-borne relapsing fever, syphilis, Q fever, bartonellosis, brucellosis, tripanosomiasis, leptospirosis, etc. [97]. In leprosy the harmful effects of dead bacteria is especially demonstrative. Single dose of 10 mg/kg rifampicin renders bacilli non-viable from 99 to 99.99% [98]; 400 mg ofloxacin or 800 mg pefloxacin kills 99.99% viable bacilli [99]. It suggests that many of the manifestations of leprosy (erythema nodosum type, nerve damage and loss of nerve function) which follow initial treatment must be due to antigens from dead organisms [100, 101]. This is an illustration that a bacterium killing often is not enough and a dead microorganism may be even more harmful than a living one.

Exotoxins are no less harmful than endotoxins. Initially it was thought that the major organisms that caused bacterial sepsis were gram-negative bacteria [102]. However, over the past 25 years it has been shown that gram-positive bacteria are the most common cause of sepsis [103]. Some of the most frequently isolated bacteria in sepsis are *Staphylococcus aureus* (*S. aureus*), *Streptococcus pyogenes* (*S. pyogenes*), *Klebsiella* spp., *Escherichia coli* (*E. coli*), and *Pseudomonas aeruginosa* (*P. aeruginosa*) [104]. Exotoxins may fatally intoxicate the host if even infection is out of the bloodstream. For example, in tetanus and diphtheria, the infection (the organism) remains localized (usually minor penetrating wounds) and the toxin is absorbed, producing major systemic effects [105, 106]. Thus, managing host intoxication by bacterial exotoxins and endotoxins is as important as killing of sepsis-causing bacteria.

### Bacterial antioxidant enzymes, hemolysins and respiration

Oxycytosis is the main mechanism of planktonic bacteria clearing from the bloodstream [22]. In oxycytosis erythrocytes “catch” bacteria by electric charge attraction forces and kill them by oxygen released from oxyhemoglobin [22]. Sepsis-causing planktonic pathogens survive oxycytosis by producing antioxidant enzymes (catalase, superoxide dismutase, glutathione peroxidase) and versatile respiration adapted to high concentrations of reactive oxygen species. Antioxidant enzymes and versatile respiration also provide bacterial survival inside erythrocytes [24].

Production of hemolysisn provides penetration of planktonic pathogens through erythrocyte membrane and forming a bacterial reservoir inside erythrocytes. Neutralization of hemolysins or inhibition of their production prevents forming of bacterial reservoirs in erythrocytes.

### Bacterial exopolymers

Encapsulated bacteria and biofilm fragments survive in the tissues and the bloodstream because of exopolymers [23]. In bacteremia exopolymers prevent oxycytosis by preventing triboelectric charging of pathogens and their attraction, fixation and oxidation on the surface of erythrocytes. Humans have no appropriate defense mechanisms for clearing encapsulated bacteria and biofilm fragments from the bloodstream. Inhibition of exopolymer production or its depolymerization may restore the effectiveness of oxycytosis and facilitate pathogen clearing from the bloodstream.

## Overcoming the problems of antibacterial therapy in sepsis

### Development of new antimicrobials

Search for new antibacterials, in particular, new antibiotics, is indispensable. Perspective antibiotics include oxazolidinones, lipopeptides, glycylcyclines, ketolides, new generations of fluoroquinolones, antistaphylococcal b-lactams, glycopeptides and others [107]. Speaking about antibiotics for sepsis therapy, the following should be taken into account: 1. Sepsis-causing bacteria enter the bloodstream as planktonic, encapsulated, L-form and biofilm fragments and a new antibacterial should be able to dissolve in bacterial polysaccharide; 2. In the bloodstream, the proliferation and the growth of sepsis-causing bacteria are inhibited by triboelectric charging (planktonic bacteria) or exopolimer insulation (encapsulated bacteria and biofilm fragments). The mechanism of action of new antibacterial medications should be different from known antibacterials and should provide killing of bacteria in the condition of low metabolic activity; 3. In the bloodstream, sepsis-causing planktonic bacteria enter erythrocytes and form bacterial reservoirs inside erythrocytes. New medications should be fat-soluble for penetrating erythrocyte membrane and accumulating inside erythrocytes; 4. New antibacterials should be beyond the mechanisms of bacterial adaptation or should affect these mechanisms, otherwise the development of bacterial resistance will continue to be a permanent problem. Three mechanisms of antimicrobial resistance predominate in bacteria: antibiotic inactivation, target site modification, and altered uptake by way of restricted entry and/or enhanced efflux [108].

### Early detection of pathogens

Early detection of pathogens and their sensitivity to bactericidal medications remain indispensable. PCR-based detection of organism [109, 110] and matrix-assisted laser desorption/ionization time-of-flight mass spectrometry (MALDI-TOF MS) [111] are becoming a helpful tool in the identification and diagnosis of blood-stream infections. Mass spectrometry also enables to distinguish drug-resistant from drug-susceptible isolates [112].

### Managing intoxication caused by endotoxins

One of basic challenges in the treatment of sepsis caused by Gram-negative bacteria is the release of endotoxin (lipopolysaccharide, LPS) from bacteria due to killing by antibiotics and/or phagocytosis in the liver and the spleen. An effective treatment must comprise the neutralization of endotoxins. LPS aggregates may interact with serum and membrane proteins such as LBP (lipopolysaccharide-binding protein) and CD14. LPS can trigger systemic hyper-inflammatory response with multiple organ failure and lethality. LPS induces inflammatory cells to express proinflammatory cytokines IL-8, IL-6, IL-1β, IL-1, IL-12, and IFNγ [113]. TNFα is of critical importance during endotoxic shock [114]. An acute exposure to endotoxin can result in life-threatening sepsis while chronic exposure has been implicated in several diverse disease states involving the gastrointestinal, nervous, metabolic, vascular, pulmonary and immune systems [115].

Humans have several mechanisms for inactivating LPS including lipid A-neutralizing proteins (bactericidal permeability-increasing protein, lactoferrin, lysozyme, collectins, etc.) [116], specific and cross-reactive anti-LPS antibodies, and sequestration of the lipid A moiety within lipoprotein micelles [117]. Intestinal alkaline phosphatase can inactivate LPS [118], but its role in LPS inactivation in humans has not been established. At present, acyloxyacyl hydrolase (AOAH) is the only endogenous enzyme known to inactivate LPS. AOAH is a 2-subunit lipase which selectively hydrolyzes the secondary (acyloxyacyl-linked) fatty acyl chains from the lipid A region of bacterial LPS. Early expectations that AOAH would protect humans from LPS-induced inflammation met with disappointment [119]. Novel types of endotoxin neutralizing compounds include peptides modified by lipophilic moieties and non-peptidic molecules, particularly lipopolyamines [120]. These peptides have been derived from bactericidal/permeability-increasing protein (BPIP), anti-microbial peptides, and leukocyte CD18 antigen [121]. Some synthetic LPS-neutralizing agents also have been developed. They include synthetic peptides, based on the endotoxin-binding domains of natural binding proteins such as lactoferrin, Limulus anti-LPS factor, NK-lysin, cathelicidins [122]. Anti-TNF antibodies have shown to help in the treatment of septic shock [123]. Endotoxin recognition molecules MD-2 and toll-like receptor 4 also may be considered as potential targets for therapeutic intervention in endotoxin shock [124]. Extracorporeal endotoxin removal or endotoxoid based vaccines have additional medical applications [120].

### Managing intoxication caused by exotoxins

A variety of gram-positive organisms are capable of causing sepsis. Those most often implicated are, in descending order of frequency, *Staphylococcus aureus, Streptococcus pneumoniae*, coagulase-negative staphylococci, beta-hemolytic streptococci, and enterococci, but virtually any gram-positive organism may be involved [125]. Gram-positive bacteria, such as *Staphylococcus aureus*, cause serious human illnesses through combinations of surface virulence factors and secretion of exotoxins. Two of the most commonly expressed superantigens (SAg), each of which has been associated with significant mortality, are staphylococcal enterotoxin B (SEB) and toxic shock syndrome (TSS) toxin–1 (TSST-1) [126]. SAgs are one of the most potent toxins produced by bacteria. They are non-glycosylated proteins that have a relatively low molecular weight [127]. SAgs are the most powerful T cell mitogens ever discovered. Concentrations of less than 0.1 pg/ml of a bacterial superantigen are sufficient to stimulate the T lymphocytes in an uncontrolled manner resulting in fever, shock and death [128]. SAgs produced by *S. pyogenes* include streptococcus pyrogenic exotoxin A and C (SPEA and SPEC) [128] and the streptococcal mitogenic exotoxin Z (SMEZ) [129]. These toxins produce a massive cellular immune response that could lead to a fatal toxic shock [130]. Classical toxic shock syndrome (TSS) caused by *S. aureus* can be considered as a capillary leak syndrome [126]. STSS, caused by *S. pyogenes*, is the most severe form of invasive streptococcal disease, with mortality rates of up to 50%. The clinical symptoms are very similar to those in TSS, but STSS is often associated with bacteraemia, myositis or necrotizing fasciitis [131]. SAgs bind to certain regions of major histocompatibility complex (MHC) class II molecules of antigen-presenting cells (APCs) and concomitantly bind to T cells. This interaction triggers the release of high amounts of various cytokines and other effectors by immune cells [132].

For developing potential therapies for conditions mediated by SAgs, toxoids have been explored [133]. In addition, monoclonal antibodies that cross-react with more than one exotoxin there have been generated [134], although it remains difficult to generate a broad-spectrum neutralizing approach because of the structural diversity of these toxins [135]. At present some different neutralizing agents against individual exotoxins have been tested and offered. Advances in selection technologies have sped up the process of generating antibodies with exquisitely tailored characteristics. In particular, synthetic antibody libraries, in which the antigen-binding sites are entirely man-made now rival or even exceed the potential of natural immune repertoires [136]. Recently, efforts have aimed to circumvent the limitations of developing antibodies in animals by developing wholly in vitro techniques for designing antibodies of tailored specificity [137]. This has been realized with the advent of synthetic antibody libraries that possess diversity outside the scope of natural immune repertoires and are thus capable of yielding specificities not otherwise attainable [137]. A number of synthetic peptides corresponding to different regions of various superantigens including SEA and TSST-1 have been studied [138, 139]. For example, it has been shown that synthetic peptide 6343 and antibody to the 6348 peptide can independently block the proliferative effects of all of the staphylococcal and streptococcal superantigens and that this inhibition is specific for the 6343 peptide, besides, the anti-peptide antibody can provide passive protection against toxic shock [140]. Synthesized α-globin chain peptides, synthetic variants of α-globin chain peptides, and two human defensins for ability to inhibit exotoxin production without significantly inhibiting *S. aureus* growth has been successfully tested [141]. Taking into account that SAgs binding to variable domains of T cell receptor beta chains (Vbeta) leads to massive release of inflammatory molecules, soluble forms of different Vbeta domains with a high affinity for binding superantigens have been generating [142]. Glycerol monolaurate (GML), a 12 carbon fatty acid monoester has been offered as a promising therapy in toxic shock syndrome. GML may reduce toxic shock mortality by suppressing TNF-alpha, *S. aureus* growth and exotoxin production [143]. Recombinant monoclonal antibodies that target staphylococcal enterotoxin B (SEB) and block receptor interactions can be of therapeutic value as well. Human monoclonal antibodies possess high affinity, target specificity, and toxin neutralization qualities essential for any therapeutic agent [144]. Intravenous polyspecific immunoglobulin G (IVIG) neutralizes the activity of a wide spectrum of superantigens [145].

### Affecting bacterial capsule

Planktonic bacteria grow and proliferate because their thin capsule does not interfere respiration and metabolic exchange. Rapidly proliferating, planktonic bacteria are short of time to produce a thick capsule. In inauspicious environment bacteria start to produce thick and viscous capsule that becomes a protective shield against environmental detrimental factors, but, at the same time, the capsule prevents nutrient/waste exchange and bacterial cells enter the phase of minimal metabolic activity. From the point of view of metabolic activity versus protection, encapsulated bacteria are between planktonic (maximal growth and proliferation/minimal protection) bacteria and bacterial spores (maximal protection/no metabolic activity). Antibacterial medications, particularly antibiotics, are not soluble in capsular polysaccharids and cannot penetrate capsule and reach bacterial wall, besides, low metabolic activity prevents absorption of antibacterial agents. On the other hand, depolymerization of bacterial capsule may re-start bacterial rapid growth and proliferation.

Capsule contains antigens and virulence factors that may be released during capsule depolymerization. Capsule polysaccharides (CPS) are not only fundamental virulence factors for a wide range of Gram-negative (e.g. *Klebsiella pneumonia*, *Escherichia coli and others*) and Gram-positive (e.g.*Streptococcus pneumonia*, *Staphylococcus aureus,* etc.) bacterial pathogens [146–148], but they also inhibit complement activity and phagocytosis [149], prevent immune recognition by antigen-specific antibodies [150] and killing by human antibody [151]. Being bacterial cell “insulator”, bacterial capsule prevents attraction, fixation and killing of bacteria by erythrocytes [23–25]. The biosynthesis of bacterial capsules is regulated by a system involving a protein tyrosine phosphatase (PTP) and a protein tyrosine kinase [152, 153]. Inhibition of these proteins may stop capsule production. As a result, bacterial virulence decreases and bacteria killing by oxidation in the bloodstream increases. Fascioquinol E inhibits PTP activity both in vitro and in vivo [154]. Capsule inhibitory drugs may become an important addition to anti-sepsis therapies.

### Affecting bacterial biofilms

In the biofilm form, bacteria are more resistant to various antimicrobial treatments, can survive harsh conditions and withstand the host’s immune system [155, 156]. Biofilm-associated infections are very difficult to treat with conventional antibiotics, therefore, the development of antibiofilm agents is indispensable. A potential antibiofilm drug that can either facilitate the dispersion of preformed biofilms or inhibit the formation of new biofilms in vivo is needed. So far, a plethora of potential antibiofilm agents with unique structures, mainly inspired by biosolutions (enzymes, phages, interspecies interactions and antimicrobial molecules from microbial origin) and natural products, have been developed and shown promise in dispersing existing biofilms or preventing bacteria from forming them [157].

The majority of the recently developed antibiofilm molecules do not directly affect bacterial survival and thus the expectation is that resistance to these molecules will not occur. It is hoped that some of these lead compounds would be translated into antibiofilm drugs [158]. To date, many antibiofilm compounds have been identified from diverse natural sources, for example, brominated furanones [159], ursine triterpenes [160], corosolic acid and asiatic acid [161], ginseng [162] and 3-indolylacetonitrile [163]. Indole, which is generated by the degradation of tryptophan by tryptophanase [164] is an intercellular signal molecule that can affect multiple aspects of some bacterial species [165] inhibiting biofilm formation and motility [166]. N-acyl homoserine lactones, cationic molecules that contain an excess of lysine and arginine residues, d**-**amino acids, monomeric trimethylsilane (TMS**),** 1-alkylquinolinium bromide ionic liquids exhibit antimicrobial and antibiofilm properties [167–171]. Nucleases such as DNase and RNase affect integrity of biofilms by degrading nucleic acid scaffold components of the extracellular matrix [172]. Nonbiocidal antibiofilm molecules for example, serine proteases, can target matrix-associated proteins [173]. Dispersin B degrades poly-*N*-acetylglucosamine (PNAG), a major polysaccharide component of many bacterial extracellular matrices [174].

The important biological messenger, nitric oxide (NO) is a signal for biofilm dispersal, inducing the transition from the biofilm mode of growth to the free swimming planktonic state. Moreover, biofilms exposed to low doses of NO are more susceptible to antimicrobial treatments than untreated biofilms [175].

Unfortunately, till now no antibiofilm drug has been registered and used in clinical practice. As a result, the treatment of biofilm-related infections is not effective. Antibiotics should be combined with antibiofilm agents. Antibiofilm agents that can both disperse and kill biofilm bacteria could have some useful applications, but remain rare [176, 177].

### Inhibition and neutralization of hemolysins

Iron is an essential nutrient for nearly all known life forms. Its capacity to readily donate or accept electrons makes it essential for important cellular redox processes [178]. Iron is the single most important micronutrient bacteria need to survive [179]. The proliferative capability of many invasive pathogens is limited by the bioavailability of iron and so pathogens have developed strategies to obtain iron from their host organisms. In turn, host defense strategies have evolved to sequester iron from invasive pathogens and human immune system has evolved ways to deprive microorganisms of this vital element [180]. During infection and inflammation, iron is withdrawn from the circulation and is redirected to hepatocytes and macrophages, thereby reducing the availability of iron to invading pathogens [181]. The ability of pathogens to acquire iron in a host is an important determinant of both their virulence and the nature of the infection produced. Bacteria utilize various iron sources which include the host proteins transferrin and lactoferrin, heme, and low molecular weight iron chelators termed siderophores [182]. Ferrous iron can also be directly imported by the G protein-like transporter, FeoB [183]. During evolution, sepsis-causing bacteria have acquired “knowledge” that iron and a rich source of protein (globin) are available in human erythrocytes and they may have access to both iron and protein by attacking and entering erythrocytes via developing and producing pore-forming enzymes (hemolysins) [24].

In sepsis, infection from the tissues enters to venous blood. Erythrocytes in the venous blood are lack of oxygen and sepsis-causing bacteria easily survive oxycytosis by producing antioxidant enzymes. Pathogens penetrate erythrocyte membrane by hemolysins and form a bacterial reservoir inside erythrocytes. High concentrations of iron inside erythrocytes are toxic for many bacteria because iron promotes the formation of damaging oxidative radicals, but sepsis-causing bacteria overcome iron toxicity by producing antioxidant enzymes. Inhibition of bacterial hemolysins may prevent pathogen penetration into erythrocytes.

Human serum lipids have inhibitory effect on staphylococcal alpha, beta and delta hemolysins, but the effect is weak [184]. *Staphylococcus aureus* self-assembling α-hemolysin heptamer is an acute virulence factor that determines the severity of *S. aureus* infections. Hence, inhibiting the heptamer formation is of considerable interest. However, both natural and chemical inhibitors reported so far has difficulties related to toxicity, bioavailability, and solubility, which necessitate in identifying some alternatives. Potential peptides for α-hemolysin inhibition was developed using in silico based approach. The peptide IYGSKANRQTDK was found to be binding efficiently with Chain A of α-hemolysin with the highest binding energy and also revealed that the designed peptide disturbed the dimer formation [185]. Totarol, a plant extract, has been revealed to inhibit the production of α-hemolysin [186]. A silkworm hemolymph protein, apolipophorin (ApoLp), binds to the cell surface of *Staphylococcus aureus* and inhibits expression of the *saePQRS* operon encoding a two-component system, SaeRS, and hemolysin genes. ApoLp bounds to lipoteichoic acids (LTA), a *S. aureus* cell surface component. These findings suggest that ApoLp binds to LTA on the *S. aureus* cell surface and inhibits *S. aureus* hemolysin gene expression via a two-component regulatory system, SaeRS [187]. The pore-forming toxin alpha-hemolysin may be also inhibited by cAMP [188].

### Inhibition of antioxidant enzymes

Antioxidant enzymes of sepsis-causing bacteria provide bacterial survival during phagocytosis in the tissues and oxycytosis (oxidation on the surface and inside erythrocytes) in the bloodstream. Catalase may function by preventing the formation of excessive concentrations of H_2_O_2_ and by using H_2_O_2_ in the peroxidatic oxidation of compounds such as methanol and formic acid. Superoxide dismutase and glutathione peroxidase play a key role in protection against radicals such as OH and O_2_^−^, and “excited” oxygen that are far more damaging for bacteria. The main reaction that glutathione peroxidase catalyzes is: 2GSH + H_2_O_2_ → GS–SG + 2H_2_O. Glutathione peroxidase catalyzes the reduction of H_2_O_2_, organic hydroperoxides, and lipid peroxides in the presence of glutathione, the hydrogen donor.. The biochemical function of glutathione peroxidase is to reduce lipid hydroperoxides to their corresponding alcohols and to reduce free hydrogen peroxide to water [189].

#### Inhibition of catalase production

Inhibition of bacterial catalase production increases the effectiveness of bacteria killing by phagocytes and erythrocytes. However, available bacterial catalase inhibitors are not safe [190–193] and new inhibitors are needed.

#### Inhibition of superoxide dismutase production

The manganese and zinc binding protein calprotectin (CP) reduces bacterial superoxide dismutase activity resulting in increased sensitivity of pathogens to oxidative stress. The inhibition of superoxide defenses by CP increases bacterial sensitivity to neutrophil-mediated killing [194, 195] and oxycytosis [22]. Bacterial MnSOD phosphorylation on serine and threonine residues decreases the bacteria capacity to counteract ROS [196].

#### Inhibition of glutathione peroxidase production

Glutathione peroxidase makes an important contribution to bacterial virulence [197–199]. It has been detected in all sepsis-causing bacteria [200, 201]. Glutathiones have relatively recently been discovered in bacteria and hence little is known about their properties. They can bind to a range of antibiotics and reduce the antimicrobial activity of *β*-lactam drugs. Understanding of antibiotic interaction with bacterial GSTs may be useful in treating bacterial resistance towards antibiotics [202]. A gene with homology to glutathione peroxidase was shown to contribute to the antioxidant defenses of *Streptococcus pyogenes* (group A streptococcus). *S. pyogenes* requires glutathione peroxidase to adapt to oxidative stress that accompanies an inflammatory response. Successful pathogens have evolved effective systems for defense against oxidative stress that include combinations of reducing enzymes, molecular scavengers, and protein and DNA repair enzymes [203, 204]. Bacterial mutants defective for resistance to oxidative stress are often avirulent [205]. Bacteria which are characterized by absence of glutathione, produce other low molecular weight thiols which fulfill the same functions as glutathione [206]. Unfortunately, at present glutathione peroxidase inhibitors are not available.

### Modulation of respiration

Sepsis-causing bacteria have flexible respiration. Being facultative anaerobes, they are the most versatile type of bacteria and can live either with or without oxygen. Sepsis-causing bacteria grow and proliferate in a certain range of respiratory activity. Bacteria better tolerate suppression of respiration than acceleration of respiration; moreover, inhibition of respiration may increase bacterial survival and tolerance to toxic agents whereas acceleration of respiration increases vulnerability of bacteria to detrimental factors. Growth inhibition from bacteriostatic antibiotics is associated with suppressed cellular respiration whereas cell death from most bactericidal antibiotics is associated with accelerated respiration [207]. In case of simultaneous action of bactericidal and bacteriostatic antibiotics, respiration deceleration provides bacterial survival. Suppression of cellular respiration by the bacteriostatic antibiotic is the dominant effect that blocks pathogen killing [207]. Inhibition of cellular respiration by knockout of the cytochrome oxidases is sufficient to attenuate bactericidal lethality whereas acceleration of basal respiration by genetically uncoupling ATP synthesis from electron transport resulted in potentiation of the killing effect of bactericidal antibiotics. Bactericidal activity can be arrested by attenuated respiration and potentiated by accelerated respiration [207]. Bacteriostatic– bactericidal combination treatments result in attenuation of bactericidal activity [208, 209]. Clinically, this effect can have negative consequences in high morbidity infections like meningitis [210], or positive effects by inhibiting lysis and exotoxin release in toxin-mediated syndromes [211]. The predominant cellular process targeted by bacteriostatic antibiotics is translation, which accounts for a major portion of the energy consumption in the cell at steady state [212, 213]. Disruption of this process may cause significant changes in cellular energy dynamics [214]. The response to bacteriostatic antibiotics may involve downregulation of major metabolic pathways [215], potentially suggesting a reduction in metabolic rates. In comparison with the bacteriostatic response, bactericidal agents may increase cellular metabolic rates and bactericidal antibiotic efficacy may be related directly to metabolic state [216]. The transcriptional response to bactericidal antibiotics involves upregulation of genes involved in central metabolism and respiration [217, 218].

At present selective accelerators and decelerators of bacterial respiration are not available and developing such agents remain a perspective field for future research.

## Bacteria killing by non-antibiotic agents

Bacterial resistance to carbapenems [219] and colistin [220] indicate that the post-antibiotic era has arrived and common infections will not be treatable with the current arsenal of antibiotics. As a result new options should be developed for treating sepsis. The use of “biological weapon” against sepsis causing bacteria is one of perspective options. It includes the use of bacteriophage, *Bdellovibrio* like organisms and Saccharomyces therapy.

### Bacteriophage therapy

The use of bacteriophages as a replacement for antibiotics in sepsis is an attractive option. Bacteriophages may be useful in the treatment of sepsis caused by antibiotic resistant bacterial infections. They have some advantages over antibiotics being more effective in treating certain infections in humans [221–224]. Phage therapy is safe and can be given intravenously in systemic infections. [225]*.* Bacterial isolates from septicemia patients spontaneously secrete phages active against other isolates of the same bacterial strain, but not to the strain causing the disease [226]. Such phages were also detected in the initial blood cultures, indicating that phages are circulating in the blood at the onset of sepsis. The fact that most of the septicemic bacterial isolates carry functional prophages suggests an active role of phages in bacterial infections [226]. Prophages present in sepsis-causing bacterial clones play a role in clonal selection during bacterial invasion [226]. The major problem of bacteriophage usage is their exquisite specificity; bacteriophages are much more specific than antibiotics. They attack only specific for them strains of bacteria, thereby precluding their use as empiric therapy in sepsis. Phage therapy is possible after identification of sepsis causing bacterium and selection of appropriate phage from existing stocks. Stocking a hospital laboratory with a complete library of phage for every conceivable bacterial pathogen is a major challenge [227]*.*

### Therapy by *Bdellovibrio* like organisms

Bdellovibrio and like organisms prey on other bacteria. They can be used as medical microbiological settings [228, 229]. *Bdellovibrio bacteriovorus* attacks a wide range of pathogens: *Escherichia coli*, *Salmonella enteric*, *Pseudomonas aeruginosa*, *S. aureus* and others [230–232].

It invades and grows within the periplasm. *Bdellovibrio bacteriovorus is* highly motile, flagellated, Gram-negative and measures 0.25 × 1.0 μm, [233]. It uses a single polar flagellum to stalk other bacteria; it burrows through the surface of its prey by secreting enzymes and consumes its host from the inside out [234, 235]. *Bdellovibrio bacteriovorus* has dual probiotic and antibiotic nature [236, 237] and it is perspective to try it in the therapy of sepsis.

### Saccharomyces therapy

Saccharomyces boulardii (SB) is a non-pathogenic yeast used in the prevention or the treatment of diarrheas [238, 239]. SB directly inhibits the growth of *Candida albicans*, *E. coli*, Shigella, *Pseudomonas aeruginosa*, *Staphylococcus aureus*, Entamoeba hystolitica, Salmonella typhimurium and Yersinia enterocolitica [240–242]. SB exerts direct anti-toxin effects and inhibits the growth of pathogens. The anti-toxin action of SB is due to a 54 kDa serine protease [243]. SB also produces a phosphatase that dephosphorylates endotoxins (such as lipopolysaccharide of *E. coli* 055B5) [244]. SB maintains epithelial barrier integrity during bacterial infection [245]. SB affects the immune response of host cells and stimulates the secretion of secretory immunoglobulin A [246]. Probably, the antimicrobial and antifungal products, produced by SB may be studied as a possible therapeutic option in sepsis.

## Bacteria clearing from the bloodstream by technical devices

Bacteria removal from the bloodstream by technical devices has a good perspective: it is effective in case of all bacterial species and does not need bacteria identification before the procedure. Plaktonic bacteria and biofilm fragments may be easily removed from the bloodstream whereas encapsulated bacteria, pathogens inside erythrocytes and bacterial L-forms may escape removal. The technical devices should be used as soon as sepsis is suspected and it should be done before empiric use of antibiotics because the latter may cause bacterial encapsulation and formation of L-forms. On the other hand, the devices provide removal and accumulation of removed bacteria in devices facilitating precise identification of pathogens.

Bacteria removal from the bloodstream was first performed 25 years ago [247]. Bacteria were removed by matrix of micro-encapsulated albumin activated charcoal (ACAC). The bacteria adhered to the ACAC, but the charcoal was not bactericidal.10 years ago for removing bacterial toxins from blood in sepsis another device was patented [248]. It includes hollow fiber that removes lipopolysaccharides (LPS) and lipoteichoic acids (LTA) from blood or plasma in an extracorporeal perfusion system.

Some years ago, for bacteria and endotoxin removing from the blood magnetic nanoparticles (MNPs) modified with bis-Zn-DPA, a synthetic ligand that binds to bacteria, was used [249]. Recently an external device that mimics the structure of a spleen and cleanses the blood in acute sepsis has been tested [250]. In this device the blood is mixed with magnetic nanobeads coated with an engineered human opsonin—mannose-binding lectin (MBL). Magnets pull the opsonin-bound pathogens and toxins from the blood then the cleansed blood is returned back to the individual. Mechanical devices can remove from the bloodstream not only bacteria, but also toxins and cytokines. For example, a mechanical devices has been developed to remove a variety of cytokines, lipopolysaccharide, or C5a from plasma [251]. A novel synthetic pyrolysed carbon monolith with controlled mesoporous domains of 2–50 nm can remove inflammatory cytokines TNF, IL-6, IL-1β and IL-8 [252]. A cytokine adsorption device (CAD) filled with porous polymer beads efficiently depletes middle-molecular weight cytokines from a circulating solution [253]. In septic patients continuous venovenous hemofiltration (CVVHF) combined with plasmapheresis (TPE) reduces mortality in single- and double-organ failure as high as 28% [254].

At present mechanical removal of pathogens and their toxins from the bloodstream by mechanical devices is the most promising clinical application that rapidly may be seen in the near future. It is most effective in case of planktonic bacteria and less effective in the removing of encapsulated bacteria and bacterial L-forms.

Antimicrobial actions needed for increasing the effectiveness of antibacterial therapy in sepsis are summarized in Table 3.

**Table 3:** Antimicrobial actions needed for increasing of sepsis therapy effectiveness

Antimicrobials	Needed actions and available agents and technologies
New antibiotics	Should be able to: a. dissolve in bacterial polysaccharides (capsule, biofilm) – not available. b. kill bacteria in the condition of low metabolic activity – not available. c. penetrate erythrocyte membrane and accumulate inside erythrocytes – not available. d. overcome bacterial adaptation and resistance – not available.
Exotoxin neutralizing compounds	Should be able to: a. cross-react with more than one exotoxin (available agents: synthetic peptide 6343 and antibody to the 6348 peptide). b. inhibit exotoxin production (available agents: synthesized α-globin chain peptides, synthetic variants of α-globin chain peptides, human defensins). c. reduce toxic shock mortality by suppressing TNF-alpha (available agent: glycerol monolaurate (GML). d. target exotoxins (available agents: recombinant monoclonal antibodies) e. neutralize the activity of superantigens (available agent: polyspecific immunoglobulin G (IVIG).
Endotoxin neutralizing compounds	Should be able to: a. neutralize endotoxins (available agents: peptides modified by lipophilic moieties and non-peptidic molecules, particularly lipopolyamines (synthetic peptides, based on the endotoxin-binding domains of natural binding proteins such as lactoferrin, Limulus anti-LPS factor, NK-lysin, cathelicidins). b. neutralize TNF (available agent: anti-TNF antibodies). c. endotoxin removal (available: extracorporeal endotoxin removal devices or endotoxoid based vaccines).
Bacterial capsule affecting agents	Should be able to: inhibit tyrosine phosphatase (PTP) and a protein tyrosine kinase (available agent: Fascioquinol E).
Bacterial biofilm affecting agents	Should be able to: a. inhibit biofilm formation and motility (available agents: brominated furanones, ursine triterpenes, corosolic acid, asiatic acid, 3-indolylacetonitrile; indole). b. exhibit antimicrobial and antibiofilm properties (available agents: N-acyl homoserine lactones, cationic molecules with an excess of lysine and arginine residues, d-amino acids, monomeric trimethylsilane (TMS), 1-alkylquinolinium bromide ionic liquids). c. affect integrity of biofilms by degrading nucleic acid scaffold components of the extracellular matrix (available agents: nucleases such as DNase and RNase). d. target matrix-associated proteins (available agents: serine proteases). e. degrade poly-*N*-acetylglucosamine (PNAG), a major polysaccharide component of many bacterial extracellular matrices (available agent: Dispersin B). f. disperse biofilm (available agent: nitric oxide (NO).
Agents that inhibit and neutralize hemolysins	Should be able to: a. inhibit the production of α-hemolysin (available agents: Totarol, cAMP). b. bound to lipoteichoic acids (available agent: apolipophorin (ApoLp).
Agents that inhibie antioxidantenzymes	Should be able to: a. inhibit superoxide dismutase (available agents: the manganese and zinc binding protein calprotectin (CP). b. inhibit catalase – not available. c. inhibit glutathione peroxidase – not available.
Agents of “Biological antibacterial weapon”	Bacteriophage therapy Therapy by *Bdellovibrio* like organisms Saccharomyces therapy
Technical devices for bacteria clearing from the bloodstream	Should be able to: a. remove bacteria and their toxins (available technologies: micro-encapsulated albumin activated charcoal (ACAC), magnetic nanoparticles (MNPs) modified with bis-Zn-DPA, bacteria binding synthetic ligands, magnetic nanobeads coated with an engineered human opsonin—mannose-binding lectin (MBL), synthetic pyrolysed carbon monolith, venovenous hemofiltration (CVVHF) combined with plasmapheresis.

## Non-antimicrobial solutions for managing sepsis

In sepsis planktonic bacteria cause abundant release of oxygen from erythrocytes [22, 23]. Oxygen oxidizes and inactivates plasma hormones and other biologically active substances. As a result, a severe endocrine dysregulation occurs in septic patients and so the replacement of hormones, peptides and other active substances in sepsis is indispensable. Corticosteroids were the first drugs tested in randomized controlled trials [255–259], then catecholamines, anti-diuretic hormone, thyroxin, insulin, adrenocorticotropin, growth hormone, estrogens, androgens, etc. were also tested [260–267]. The results of separate and combined use of hormones are controversial and the positive effect is not convincing. Hormonal replacement therapy (protocol) should include simultaneous use of a combination of hormones that takes into account their synergism and antagonism, anabolic and catabolic properties, half-life, resistance to oxidation, pharmacokinetics, pharmacodynamics, etc. The profile and proportions of most important hormones and regulatory substances for support of vital functions should be established and the replacement of all indispensable hormonal and other regulatory components should be performed. Injected components may be oxidized and inactivated so constant control of their concentrations is necessary.

## Optimal route and timing of antibiotic administration in sepsis

Central venous catheters (CVC) are an integral part in medical management of sepsis, particularly, they are indispensable for antibiotic therapy. In sepsis catheters can be placed in veins in the neck (internal jugular vein), chest (subclavian vein or axillary vein), groin (femoral vein), or through veins in the arms (a PICC line, or peripherally inserted central catheters). Catheters are used to administer medication or fluids that are unable to be taken by mouth or would harm a smaller peripheral vein, obtain blood tests (specifically the “central venous oxygen saturation”), measure central venous pressure, etc. Three anatomical sites (the subclavian, jugular, or femoral vein) are commonly used to insert central venous catheters, but insertion at each site has the potential for major complications. Subclavian-vein catheterization is associated with a lower risk of bloodstream infection and symptomatic thrombosis and a higher risk of pneumothorax than jugular-vein or femoral-vein catheterization [268].

Subclavian and internal jugular CVC have similar risks for catheter-related complications in long-term catheterization. Subclavian CVC is preferable to femoral CVC in short-term catheterization because of lower risks of catheter colonization and thrombotic complications. In short-term catheterization, femoral and internal jugular CVA routes have similar risks for catheter-related complications; internal jugular CVA routes are associated with higher risks of mechanical complications [269].

In sepsis pathogens circulate in the bloodstream. Catheters themselves can introduce bacteria into the bloodstream. Catheter-related bloodstream infections (CRBSIs) may deteriorate the condition of patients with sepsis. Although earlier studies showed a lower risk of catheter-related bloodstream infections when the internal jugular was compared to the femoral site, recent studies show no difference in the rate of catheter-related bloodstream infections between the sites [270]. Biofilms that harbor microorganisms are demonstrated on external and internal surfaces of the indwelling catheters within as early as 24 h after their placement [271].

If a central line infection is suspected in a person, blood cultures are taken from both the catheter and a vein elsewhere in the body. If the culture from the central line grows bacteria much earlier (> 2 h) than the other vein site, the line is likely infected. Quantative blood culture is more accurate, but it is not widely available [272].

To prevent infection, stringent cleaning of the catheter insertion site is advised. Povidone-iodine solution is often used for such cleaning, but chlorhexidine is twice as effective as iodine [273]. Routine replacement of lines makes no difference in preventing infection [274]. Recommendations regarding risk reduction for infection of CVCs, include antibiotic lock therapy - a method for sterilizing the catheter lumen that involves instilling high concentrations of antibiotics into the catheter lumen for extended periods of time. Results from in vitro studies demonstrate stability of antibiotics while maintaining high concentrations for prolonged periods of time. In vivo studies show antibiotic lock technique as an effective and safe option for both prevention and treatment of CRBSIs [275]. Recently, non-antibiotic antimicrobial catheter lock solutions also are used [276].

Sepsis starts when infection enters the bloodstream and overcomes the host mechanisms of blood clearing from bacteria. The most common primary sites of infection include the lungs, urinary tract, abdominal organs, and pelvis. Early source identification is important if sepsis is to be treated adequately. Empiric antimicrobial therapy is the cornerstone of the treatment [6]. Before giving antibiotics, blood cultures should be taken. Blood culture provides information regarding the infection and bacteria sensitivity to antibiotics. Revealing the source of infection is necessary for targeting of antibiotics. The primary site of infection may be the source of constant bacteremia during the course of sepsis. The blood culture may help to choose appropriate antibiotics and de-escalate from broad spectrum to narrow spectrum antimicrobials. Although blood cultures are the gold standard in identifying infections, other interventions may be also needed.

Current guidelines recommend starting antibiotic therapy in sepsis as early as possible and within one hour of identification of septic shock [7]. The Surviving Sepsis Campaign (SSC) published their initial clinical practice guidelines (CPG) for the management of severe sepsis and septic shock in 2004 [8]. Updated versions were published in 2008 [277], 2012 [11] and most recently in 2016 [278] and 2018 [279]. The Surviving Sepsis Campaign bundle is the core of the Campaign**’**s quality improvement efforts. Applying the sepsis bundle simplifies the complex processes of the care of patients with sepsis. The “sepsis bundle” has been central to the implementation of the Surviving Sepsis Campaign from the first publication of its evidence-based guidelines in 2004 through subsequent editions. Тhe bundle elements were designed to be updated as indicated by new evidence and have evolved accordingly. Updates to clinical management guidelines precede the updates to the sepsis bundles. The first sepsis bundle published in 2004 included a “Sepsis Resuscitation Bundle” to be completed “as soon as possible” within the first 6 h of presentation and a “Sepsis Management Bundle” to be completed “as soon as possible” within the first 24 h [8]. These initial bundles were revised in 2012 and changed to a “3-h bundle” and “6**-**h bundle,” with similar elements but an effort to perform the interventions within a shorter time period [11]. These 3- and 6-h bundles were further revised in 2015 with the elimination of central venous pressure (CVP) and S_CV_O_2_ measurement. Driven by the release of the International Guidelines for Management of Sepsis and Septic Shock: 2016 (guideline summary), a new bundle update was published in 2018 titled “The Surviving Sepsis Campaign Bundle: 2018 Update.” [279]. The most important change in this new revision of the SSC bundles is that the 3-h and 6-h bundles have been combined into a single “hour-1 bundle” with the explicit intention of beginning resuscitation and management immediately. The Hour-1 bundle should be viewed as a quality improvement opportunity moving toward an ideal state. For critically ill patients with sepsis or septic shock, time is of the essence. Although the starting time for the Hour-1 bundle is recognition of sepsis, both sepsis and septic shock should be viewed as medical emergencies requiring rapid diagnosis and immediate intervention [279]. The Hour-1 bundle encourages clinicians to act as quickly as possible to obtain blood cultures, administer broad spectrum antibiotics, start appropriate fluid resuscitation, measure lactate, and begin vasopressors if clinically indicated. Ideally these interventions would all begin in the first hour from sepsis recognition but may not necessarily be completed in the first hour. Minimizing the time to treatment acknowledges the urgency that exists for patients with sepsis and septic shock. The new “Hour-One Bundle” includes 5 steps which are recommended to begin immediately upon presentation in all patients with clinical elements suspicious for sepsis or septic shock (Table 4).

**Table 4:** Surviving sepsis campaign hour-1 bundle of care elements

• Measure lactate level^a^ • Obtain blood cultures before administering antibiotics. • Administer broad-spectrum antibiotics. • Begin rapid administration of 30 ml/kg crystalloid for hypotension or lactate level ≥ 4 mmol/L. • Apply vasopressors if hypotensive during or after fluid resuscitation to maintain MAP ≥65 mmHg.	

^a^Remeasure lactate if initial lactate is elevated (> 2 mmol/L)

Questions about the use of triage time in the emergency department as “time zero” for starting the clock to score compliance with the elements of the Surviving Sepsis Campaign (SSC) bundles have been raised since the bundles’ 2005 introduction as a performance improvement tool. “Time zero” is the time of presentation to triage in the emergency department or if presentation occurs in a different setting (outpatient, nursing home, intensive care unit, hospital floor). “Time zero” would be the first documentation in the chart with the elements of sepsis [279]. It is understood that the interventions may not be completed within the hour**.** At the same time in sepsis every hour delay is associated with a 6% rise in mortality [8, 9]. There are no prospective data that early broad-spectrum antibiotic therapy reduces mortality in severe sepsis, but prompt initiation of antimicrobial therapy remains important for suspected infections [10]. If the pathogen is resistant to antibiotic, early or late initiation of antibiotic therapy cannot improve the outcome. Inappropriateness of empirical antibiotic therapy can contribute to high level of mortality [11]. The 2016 guidelines recommend administering empiric broad-spectrum antimicrobials that cover all likely pathogens [278]. The initial empiric antibiotic regimen for patients in septic shock should include at least two antibiotics from different classes (combination therapy) directed toward the most likely pathogens. Treatment should be narrowed once the pathogen and its antimicrobial sensitivities are ascertained or when the patient demonstrates clinical improvement. With respect to antibiotic duration, combination therapy in patients with septic shock should be de-escalated to monotherapy within a few days if clinical improvement or with evidence of infection resolution [278]. Total treatment duration should be 7–10 days for infections with sepsis or septic shock; however, some patients may warrant a prolonged course if they respond slowly to treatment, do not have source control, have bacteremia with *Staphylococcus aureus* or have immunological deficiencies or fungal/viral infections [278].

## Conclusion

Bacteria cause sepsis being in different forms: planktonic, encapsulated, L-form and biofilm fragments. Antibacterial therapy is most effective when infection is in the tissues. If infection enters the bloodstream and starts to occupy erythrocytes, the effectiveness of antibacterial therapy dramatically decreases. So the most effective approach to sepsis treatment is prevention of bacteremia. Sublethal effect of antibacterial drugs in the tissues may provoke bacterial encapsulation, biofilm growth, switching to L-form. Early detection of infection in the tissues and selection of appropriate antibacterial medication in adequate doses is of great importance. Inhibition of the production of bacterial antioxidant enzymes (catalase, superoxide dismutase, glutathione peroxidase) may increase the effectiveness of phagocytosis in the tissues and oxycytosis in the bloodstream. Inactivation of bacterial hemolysins may prevent bacterial penetration through erythrocyte membranes and forming of infection reservoir inside erythrocytes. Acceleration of bacterial respiration may increase the effectiveness of bactericidal drugs. Dispersion of bacterial exopolymers is indispensible in antibacterial therapy of infection caused by encapsulated bacteria and biofilm. Inhibition, inactivation or binding of bacterial LPS and SAgs is necessary for preventing of host intoxication and decreasing of infection virulence.

Sepsis therapy should include the use of antibacterial medications, modulation of bacterial respiration, inhibition of bacterial antioxidant enzymes and hemolysins, neutralization of exo- and endotoxins, dispersion of bacterial capsule and biofilm, increasing of host tolerance to bacterial products, facilitation of host bactericidal mechanisms, support of host vital functions and restore of homeostasis.
